# Sleeve gastrectomy improves lipid dysmetabolism by downregulating the USP20-HSPA2 axis in diet-induced obese mice

**DOI:** 10.3389/fendo.2022.1041027

**Published:** 2022-12-27

**Authors:** Wenjie Zhang, Bowen Shi, Shirui Li, Zenglin Liu, Songhan Li, Shuohui Dong, Yugang Cheng, Jiankang Zhu, Guangyong Zhang, Mingwei Zhong

**Affiliations:** ^1^ Department of General Surgery, Shandong Provincial Qianfoshan Hospital, Shandong University, Jinan, China; ^2^ Cheeloo College of Medicine, Shandong University, Jinan, China; ^3^ Department of General Surgery, The First Affiliated Hospital of Shandong First Medical University, Jinan, China

**Keywords:** sleeve gastrectomy, lipid dysmetabolism, USP20, HSPA2, diet-induced obese

## Abstract

**Introduction:**

Obesity is a metabolic disease accompanied by abnormalities in lipid metabolism that can cause hyperlipidemia, non-alcoholic fatty liver disease, and artery atherosclerosis. Sleeve gastrectomy (SG) is a type of bariatric surgery that can effectively treat obesity and improve lipid metabolism. However, its specific underlying mechanism remains elusive.

**Methods:**

We performed SG, and sham surgery on two groups of diet-induced obese mice. Histology and lipid analysis were used to evaluate operation effect. Immunohistochemistry, immunoblotting, real-time quantitative PCR, immunoprecipitation, immunofluorescence and mass spectrometry were used to reveal the potential mechanisms of SG.

**Results:**

Compared to the sham group, the SG group displayed a downregulation of deubiquitinase ubiquitin-specific peptidase 20 (USP20). Moreover, USP20 could promote lipid accumulation *in vitro*. Co-immunoprecipitation and mass spectrometry analyses showed that heat-shock protein family A member 2 (HSPA2) potentially acts as a substrate of USP20. HSPA2 was also downregulated in the SG group and could promote lipid accumulation *in vitro*. Further research showed that USP20 targeted and stabilized HSPA2 via the ubiquitin-proteasome pathway.

**Conclusion:**

The downregulation of the USP20-HSPA2 axis in diet-induced obese mice following SG improved lipid dysmetabolism, indicating that USP20-HSPA2 axis was a noninvasive therapeutic target to be investigated in the future.

## Introduction

Obesity has become a global public health concern, placing a high disease burden on society (1). Obesity causes abnormal lipid metabolism, which results in hyperlipidemia, non-alcoholic fatty liver disease, and artery atherosclerosis (2). Bariatric surgery can effectively reduce body weight and obesity-related complication risks in patients with morbid obesity (3). There are several types of bariatric surgeries, however, the sleeve gastrectomy (SG) ranks among the most frequently performed procedures (4). Several studies have observed that SG contributed to weight loss and improved lipid metabolism in human and animal models (5–8). However, the mechanism by which SG improves lipid metabolism remains elusive.

In eukaryotic cells, ubiquitination plays an important role in post-translational modification (9). Deubiquitination is the opposite of ubiquitination, and both processes are always in a dynamic equilibrium (10). When proteins are marked by ubiquitin chains, the proteasome targets and degrades the ubiquitinated proteins (11). Deubiquitinases (DUB) in the deubiquitination process remove ubiquitin chains to preserve the labeled substrate proteins (12). Ubiquitin-specific peptidase 20 (USP20) is a pivotal member of the DUB family and regulates the stability of multiple proteins *via* the ubiquitin-proteasome pathway (13, 14). USP20 is also associated with multiple biological processes (15–19). Emerging studies and trials have revealed the important role of USP20 in improving lipid metabolism and the treatment of metabolic diseases, including obesity, hyperlipidemia, hepatic steatosis, and diabetes (20). Whether USP20 plays a role in lipid metabolism homeostasis mediated by SG is unknown.

Heat-shock protein family A member 2 (HSPA2) is a member of the evolutionarily conserved heat-shock protein chaperone family (21). HSPA2 participates in spermatogenesis and was originally recognized as a testis-specific chaperone protein (22). Subsequent research has shown that HSPA2 gene polymorphisms are highly correlated with obesity, where individuals with the homozygous genotype are susceptible to obesity, suggesting that HSPA2 may play a pivotal role in the initiation and progression of obesity and related metabolic disorders (23).

This study aimed to investigate the underlying mechanisms of SG as a treatment for obesity in improving lipid metabolism on diet-induced obese (DIO) mice.

## Materials and methods

### Animals

**Figure 1:**
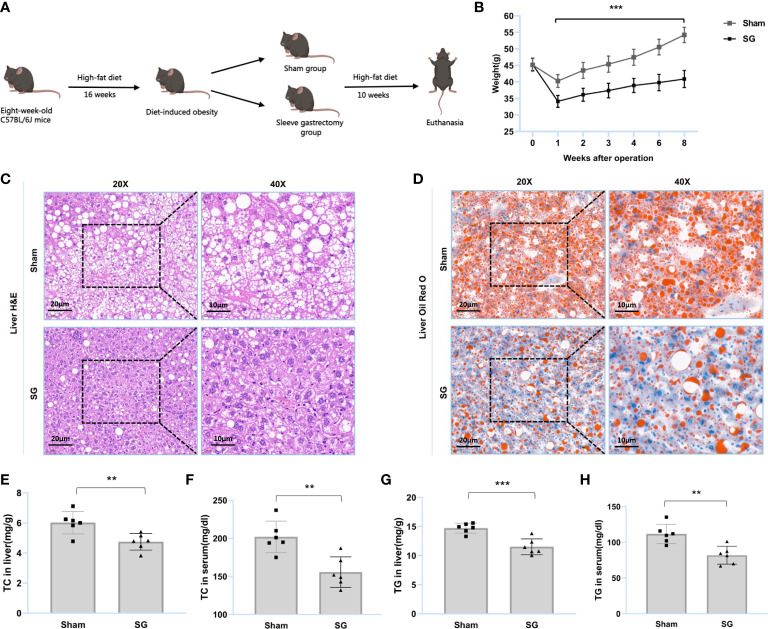
SG improves lipid dysmetabolism of DIO mice. **(A)** Flow diagram of SG model establishment in DIO mice. **(B)** Body weight after surgery (n = 6). **(C, D)** H&E and Oil Red O staining of liver tissue from sham and SG group. Scale bars were shown in the figure. **(E, F)** Liver and serum TC levels of sham and SG group (n = 6). **(G, H)** Liver and serum TG levels of sham and SG group (n = 6). The data are expressed as mean ± SEM, Student’s t-test was used for intergroup comparisons. **p < 0.01, ***p < 0.001.

Eight-week-old C57BL/6J male mice were purchased from Weitong Lihua Experimental Animal Technology. They were kept under 12-hour light and dark cycles at 22°C, with *ad libitum* access to normal food and water. After a week of adaptive feeding, the mice were fed an *ad libitum* high-fat diet (HFD) for 16 weeks to induce obesity (n = 25). Mice with body weights in the range of 42–48 g were considered as successful DIO models (n = 17). Thereafter, the obese mice were divided into two groups. One group underwent SG (SG group = 10), and the other group underwent sham surgery (sham group = 7). After surgery, the mice were fed the same HFD as during pre-operation for 10 weeks. Four SG mice died from complications of surgery and one sham mouse was excluded because of vision loss. Finally, six SG and six sham mice were included in the analyses (**Figure 1A**). All protocols for animal experiments were approved by the Medical Ethics Committee of Shandong Provincial Qianfoshan Hospital, Shandong University, and all animal studies complied with relevant ethical regulations for animal testing and research.

### Surgical procedures

Before surgery, C57BL/6J mice were fed Ensure (Abbott, USA) for 2 d and were subsequently fasted overnight. SG and sham surgery were performed under anesthesia with 2% isoflurane. The lateral 80% of the stomach was excised to reform a tubular stomach. The cardia and pylorus were retained. The procedure of sham included analogous isolation of the stomach and manual pressure along a vertical line between the esophageal sphincter and the pylorus with blunt forceps (24). After surgery, mice were single-housed and their diet was gradually advanced to an HFD, which was consistent with the preoperative diet. Body weight changes were recorded weekly, and the animals were euthanized 10 weeks after surgery.

### Histology, immunohistochemistry, and lipid analysis

Frozen or paraffin sections were used for histology and immunohistochemistry. Fresh frozen sections were used for Oil Red O staining to evaluate lipid content. Moreover, formalin-fixed paraffin-embedded sections were prepared for hematoxylin and eosin (H&E) staining and immunohistochemistry analysis. For immunohistochemistry, antigen retrieval was performed using heated citrate buffer (Solarbio, China) before blocking endogenous peroxidase activity. After blocking with 10% goat serum, the sections were incubated with primary antibodies against USP20 (ProteinTech, 1:200) or HSPA2 (ProteinTech, 1:200) overnight at 4°C. Thereafter, the sections were incubated with HRP-conjugated anti-rabbit/mouse IgG for 0.5 h at 37°C. The color was developed using 3,3′-diaminobenzidine (Beyotime, China), and the nuclei were stained with hematoxylin (Beyotime). Three images from each section were captured using a light microscope (Olympus, Japan). Additionally, total cholesterol (TC) and triglyceride (TG) levels in the serum and liver were measured using an enzymatic assay kit (Nanjing Jiancheng, China), according to the manufacturer’s protocol.

### Cell cultures and treatments

HepG2 human hepatocellular carcinoma cells and human embryonic kidney 293T (HEK-293T) cells were purchased from the Chinese Academy of Sciences Cell Bank (Shanghai, China). Short tandem repeat profiling was used to validate the identities of all cell lines. HepG2 and HEK-293T cells were cultured in DMEM/HIGH GLUCOSE medium (Gibco, USA) containing 10% fetal bovine serum (FBS; Gibco, USA). The incubator conditions were set at 37°C, 5% CO_2_. Palmitic acid (Sigma-Aldrich, Germany) was added to the culture medium to establish a lipid-loaded cell model *in vitro*, which was used to mimic an *in vivo* high-fat environment.

### Oil red O staining and lipid quantification

HepG2 human hepatocellular carcinomas cells were washed three times with phosphate-buffered saline (PBS), and subsequently fixed with 4% paraformaldehyde for 0.5 h. Fixed cells were washed with 60% isopropanol and stained for 0.5 h in Oil Red O solution. Before nuclear counterstaining with hematoxylin, cells were washed again with 60% isopropanol. Finally, three images of each sample were captured using a light microscope (Olympus). To quantify the lipid content, 100% isopropanol was used to extract the Oil Red O stained lipid droplets, and the absorbance was measured at 495 nm.

### Antibody, immunoblotting, immunoprecipitation, and immunofluorescence

The primary antibody information is summarized in **Supplementary Table S1**. Immunoblotting, IP, and IF were performed as previously described (25, 26). Briefly, a RIPA buffer containing a protease inhibitor mixture (Beyotime) and phosphatase inhibitor cocktail (Beyotime) was used to harvest and extract total proteins. A BCA kit was used to determine the protein concentration. Total proteins were separated using 8%, 10%, and 15% SDS-polyacrylamide gel electrophoresis (PAGE), and subsequently transferred onto a polyvinylidene difluoride membrane (Millipore, Germany). Proteins were incubated overnight with the indicated antibodies. After incubation, the signal was detected using electrochemiluminescence reagents (Thermo Scientific). For IP, total proteins were incubated with the indicated antibodies for 4–6 h to form protein-antibody complexes. Thereafter, the protein-antibody complexes were incubated with protein A/G magnetic beads (Santa Cruz Biotechnology, USA) for 16–20 h at 4°C. The mixture was subsequently centrifuged at 1000 rpm for 5 min to collect immunoprecipitants. The immunoprecipitants were washed five times with lysis buffer before solubilization in sample buffer and analysis using SDS-PAGE. For IF, cells were fixed with 4% paraformaldehyde and blocked with goat serum. Thereafter, cells were incubated with the primary antibody overnight and subsequently washed three times with PBS. Fluorescent secondary antibody incubation was performed before nuclear staining with 4’, 6-diamidino-2-phenylindole (Beyotime). The samples were analyzed using a fluorescence microscope (Olympus).

### Real-time quantitative PCR

Total RNA was extracted from cells and tissues using RNA extraction kits (Feijie, China) according to the PrimeScript RT Master Mix (Takara, Japan) with the manufacturer’s cDNA. Real-time quantitative PCR was conducted using FastStart Essential DNA Green Master (Roche, Switzerland) on a Roche LightCycler 480 (Roche). The expression values for the indicated genes were normalized to those of β-actin. The primers were synthesized by the Shenzhen Huada Gene Company and are presented in **Supplementary Table S2**.

### Mass spectrometry based non-targeted proteomics

According to the manufacturer’s protocol, gel pieces were digested overnight using trypsin at 37°C. The mass spectrometer type was Thermo Scientific™ Q Exactive Plus. The tryptic peptides flowed at a constant rate of 400 nL/min on an EASY-nLC 1000 UPLC system, and the Orbitrap detected intact peptides and fragments at a resolution of 70,000 and 17,500, respectively. It was a data-dependent procedure that alternated between one MS scan followed by 20 MS/MS scans with a 15.0 s dynamic exclusion. Automatic gain control was set at 5E4. Proteome discoverer 1.3 was used to process the resulting data, and tandem MS spectra were performed against the SwissProt human database (20387 sequences). Trypsin was specified as a cleavage enzyme that allowed up to two missing cleavages. Mass error was set to 10 ppm for precursor ions and 0.02 Da for fragment ions. Carbamidomethyl on Cys was specified as fixed modification and oxidation on Met was specified as variable modification. The peptide confidence was high, and the peptide ion score was >20.

### DNA constructs and transfection

Flag-USP20 and Myc-HSPA2 cDNA, USP20 and HSPA2 short hairpin RNA (shRNA) lentiviral constructs, and corresponding controls were purchased from ViGene Biosciences (Shandong, China). DNA sequencing was performed to verify the above plasmids using Invitrogen Biotechnologies (Shanghai, China). **Supplementary Table S3** provides detailed information on DNA constructs. Transfection was performed using Lipofectamine™ 2000 (Invitrogen, USA) according to the manufacturer’s instructions.

### Cycloheximade analyses and *in vivo* ubiquitination

For CHX determination, cells were treated with 100 µg/mL CHX before collection for immunoblotting at the indicated time. For *in vivo* ubiquitination, expression plasmids encoding Myc-HSPA2, HA-ubiquitin, and Flag-USP20 were used to transfect the cells alone or in combination. Twenty-four hours after transfection, cells were treated with MG-132 and lysed for immunoblotting and IP analysis.

### Statistical analysis

Immunoblotting statistics were performed using ImageJ 1.53e. The standard error of the mean (SEM) was defined as the present form for all data. An unpaired two-tailed Student’s *t*-test was used for intergroup comparisons using GraphPad Prism 9. Statistical significance was set at *p <*0.05.

## Results

### SG improves lipid dysmetabolism of DIO mice

SG was found to improve lipid dysmetabolism in DIO mice. The main process of the trial is summarized in **Figure 1A**. The results showed that the body weights of the SG group were significantly lower than those of the sham group (**Figure 1B** and **Supplementary figure S1**). H&E staining showed that SG reduced the number of ballooning hepatocytes and attenuated hepatic steatosis (**Figure 1C**). Oil Red O staining showed that SG attenuated DIO hepatic lipid accumulation (**Figure 1D**). Furthermore, the TC and TG levels in the liver and serum of SG group individuals were significantly reduced (**Figures 1E-H** and **Supplementary Figures S2, S3**). These results suggested that SG can improve lipid metabolism in DIO mice.

### USP20 promotes lipid accumulation *in vitro*

**Figure 2:**
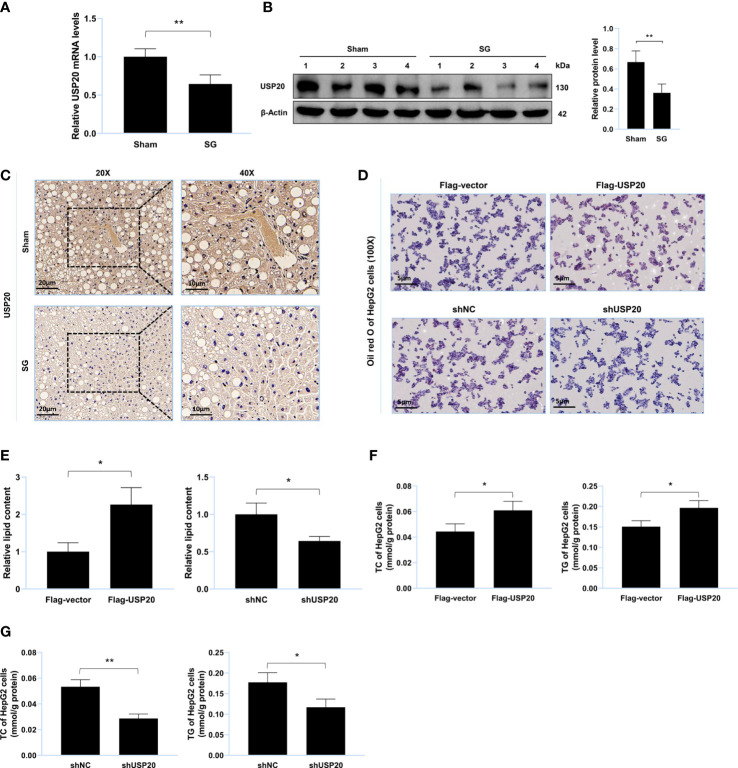
USP20 promotes lipid accumulation *in vitro*. **(A)** Relative mRNA level of USP20 in liver from sham group and SG group (n = 4). **(B)** Western blot detection of USP20 in liver from sham group and SG group (n = 4). **(C)** Immunohistochemistry detection of USP20 in liver from sham group and SG group. Scale bars were shown in the figure. **(D)** HepG2 cells were transfected with Flag-USP20, Flag-vector, shUSP20 and shNC. Lipid accumulation of HepG2 cells was assessed by Oil Red O staining. Scale bars were shown in the figure. **(E)** Relative lipid content of HepG2 cells (n = 3). **(F, G)** TC and TG content of HepG2 cells (n = 3). The data are expressed as mean ± SEM, Student’s t-test was used for intergroup comparisons. *p < 0.05, **p < 0.01.

To explore whether USP20 was involved in the improvement of lipid metabolism after surgery, we first investigated its expression in the mouse liver. Compared to those of the sham group, both the mRNA and protein levels of USP20 in the SG group were downregulated (**Figures 2A–C**), implying that USP20 might be involved in the regulation of lipid metabolism. To verify whether USP20 promotes lipid accumulation, Oil Red O staining of lipid-loaded HepG2 cells was performed, which showed that USP20 overexpression could promote lipid accumulation (**Figure 2D**) while USP20 knockdown attenuates it (**Figure 2E**). Moreover, USP20 overexpression resulted in significantly higher levels of TC and TG (**Figure 2F**), whose accumulations were significantly reduced under USP20 knockdown (**Figure 2G**). Our research demonstrated that SG downregulates the expression level of USP20, which attenuates lipid accumulation *in vitro*.

### HSPA2 promotes lipid accumulation *in vitro*

**Figure 3:**
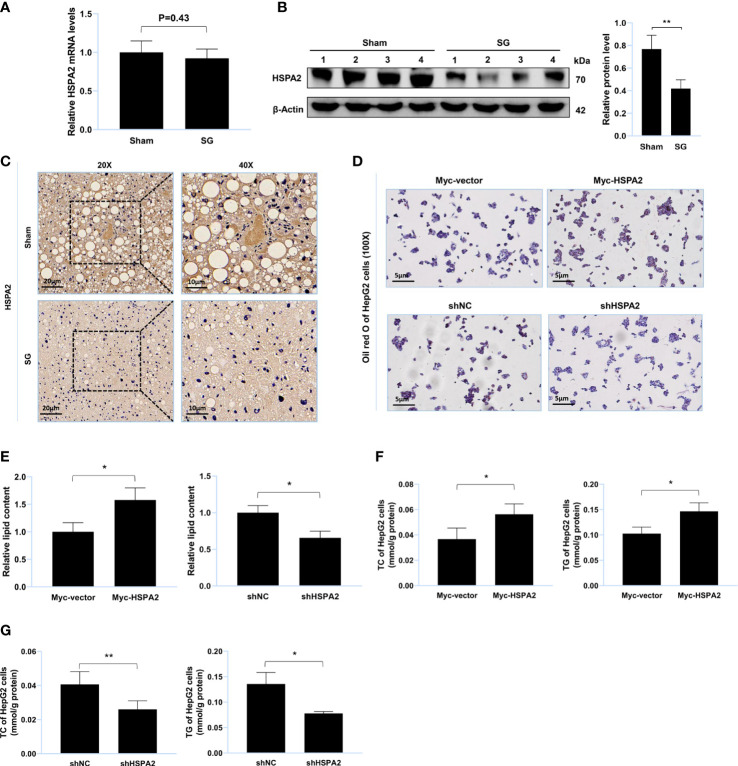
HSPA2 promotes lipid accumulation *in vitro*. **(A)** Relative mRNA level of HSPA2 in liver from sham group and SG group (n = 4). **(B)** Western blot detection of HSPA2 in liver from sham group and SG group (n = 4). **(C)** Immunohistochemistry detection of HSPA2 in liver from sham group and SG group. Scale bars were shown in the figure. **(D)** HepG2 cells were transfected with Myc-HSPA2, Myc-vector, shHSPA2 and shNC. Lipid accumulation of HepG2 cells was assessed by Oil Red O staining. Scale bars were shown in the figure. **(E)** Relative lipid content of HepG2 cells (n = 3). **(F, G)** TC and TG content of HepG2 cells (n = 3). The data are expressed as mean ± SEM, Student’s t-test was used for intergroup comparisons. *p < 0.05, **p < 0.01.

MS was used to identify USP20 substrates (**Supplementary Table S4**) and HSPA2 was identified as a potential substrate. Thereafter, we researched the expression level of HSPA2 in the mouse liver. Compared to the sham group, the protein level of HSPA2 and not mRNA, was downregulated in the SG group (**Figures 3A–C**), implying that HSPA2 may regulate lipid metabolism. Oil Red O staining of the lipid-loaded cell model showed that HSPA2 overexpression promoted lipid accumulation (**Figure 3D**), while HSPA2 knockdown attenuated lipid accumulation (**Figure 3E**). Similarly, HSPA2 overexpression resulted in significantly higher levels of TC and TG (**Figure 3F**), while HSPA2 knockdown significantly reduced the accumulation of TC and TG (**Figure 3G**). The above results showed that SG downregulates the expression level of HSPA2, and that HSPA2 also promotes lipid accumulation *in vitro*.

### USP20 targets and stabilizes HSPA2 at the protein level

**Figure 4:**
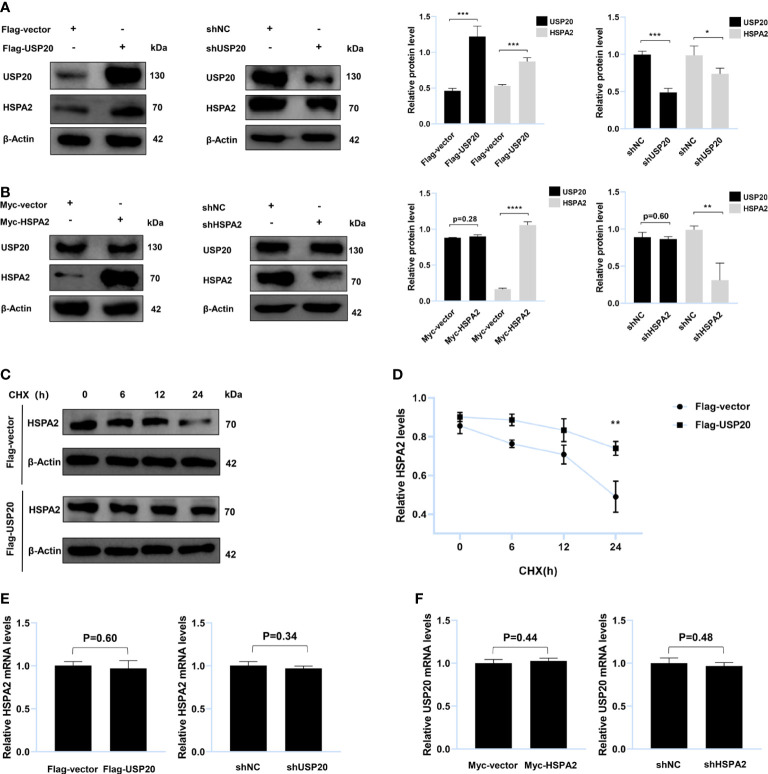
USP20 targets and stabilizes HSPA2 at protein level. **(A)** Immunoblotting analysis of HepG2 cells stably expressing Flag-USP20, Flag-vector, shUSP20 and shNC with the indicated antibodies (n = 3). **(B)** Immunoblotting analysis of HepG2 cells stably expressing Myc-HSPA2, Myc-vector, shHSPA2 and shNC with the indicated antibodies (n = 3). **(C)** Transfection with the indicated expression vectors was performed in HepG2 cells. After 24h, 100μg/ml of CHX was used to treat HepG2 cells for the indicated times and then analyzed by immunoblotting with the indicated antibodies (n = 3). **(D)** Quantitative results of HSPA2 protein levels (HSPA2/β-actin, n = 3). **(E)** PCR analysis of HepG2 cells stably expressing Flag-USP20, Flag-vector, shUSP20 and shNC with the indicated primers (n = 3). **(F)** PCR analysis of HepG2 cells stably expressing Myc-HSPA2, Myc-vector, shHSPA2 and shNC with the indicated primers (n = 3). The data are expressed as mean ± SEM, Student’s t-test was used for intergroup comparisons. *p < 0.05, **p < 0.01, ***p < 0.001 and ****p < 0.0001.

To verify whether USP20 targeted HSPA2, HepG2 cells were transfected with Flag-USP20 cDNA and USP20 short hairpin RNA. The results indicated that overexpression and knockdown of USP20 caused respective upregulation and downregulation of HSPA2 at the protein level (**Figure 4A**). However, no significant change in USP20 was observed upon cell transfection with Myc-HSPA2 cDNA and HSPA2 short hairpin RNA (**Figure 4B**). Cells were subsequently treated with CHX, and we found that overexpression of USP20 significantly delayed the degradation of HSPA2 (**Figures 4C, D**). Real-time quantitative PCR showed that there was no reciprocal regulation between USP20 and HSPA2 at the mRNA level (**Figures 4E, F**). These results suggested that USP20 targets and stabilizes HSPA2 at the protein level.

### USP20 stabilizes HSPA2 *via* deubiquitination

**Figure 5:**
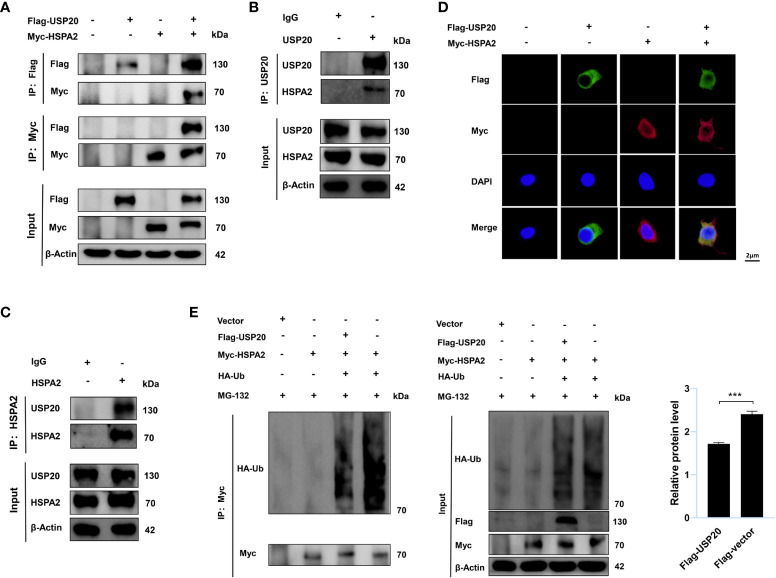
USP20 stabilizes HSPA2 via deubiquitination. **(A)** After 48h transfection of HEK293T cells with the indicated expression vectors, cellular lysates were analyzed by IP and immunoblotting with the indicated antibodies. **(B, C)** The lysates of HEK293T cells were analyzed by IP and immunoblotting with the indicated antibodies. **(D)** Transfection with the indicated expression vectors was performed in HEK293T cells. And 48h after transfection, indirect IF staining was performed with the indicated antibodies. Nuclear staining was performed with 4’, 6-diamidino-2-phenylindole. Scale bars were shown in the figure. **(E)** Transfection with the indicated expression vectors was performed in HEK293T cells. After 48h, cells were treated with MG-132 (10μM) for 6h and then analyzed by IP and immunoblotting with the indicated antibodies. Three times experiments were performed for above results. The data are expressed as mean ± SEM, Student’s t-test was used for intergroup comparisons. ***p < 0.001.

To gain insight into the USP20-HSPA2 axis mechanism, we first examined whether there was an interaction between USP20 and HSPA2. Immunoblotting suggested that co-immunoprecipitation occurred when Flag-USP20 and Myc-HSPA2 were co-expressed in HEK-293T cells (**Figure 5A**). The endogenous IP showed similar results (**Figures 5B, C**). Moreover, IF analysis showed that USP20 co-localized with HSPA2 in the cytoplasm of HEK-293T cells (**Figure 5D**). These results indicated an interaction between USP20 and HSPA2, and owing to the deubiquitinating enzyme function of USP20, we tested whether USP20 induced the deubiquitination of HSPA2. As shown in **Figure 5E**, Myc-HSPA2 was ubiquitinated in the presence of HA-ubiquitin, and the co-expression of Flag-USP20 reduced the ubiquitination of Myc-HSPA2. Our results therefore demonstrated that the mechanism of the USP20-HSPA2 axis involves the stabilization of HSPA2 by USP20 *via* deubiquitination.

**Figure 6:**
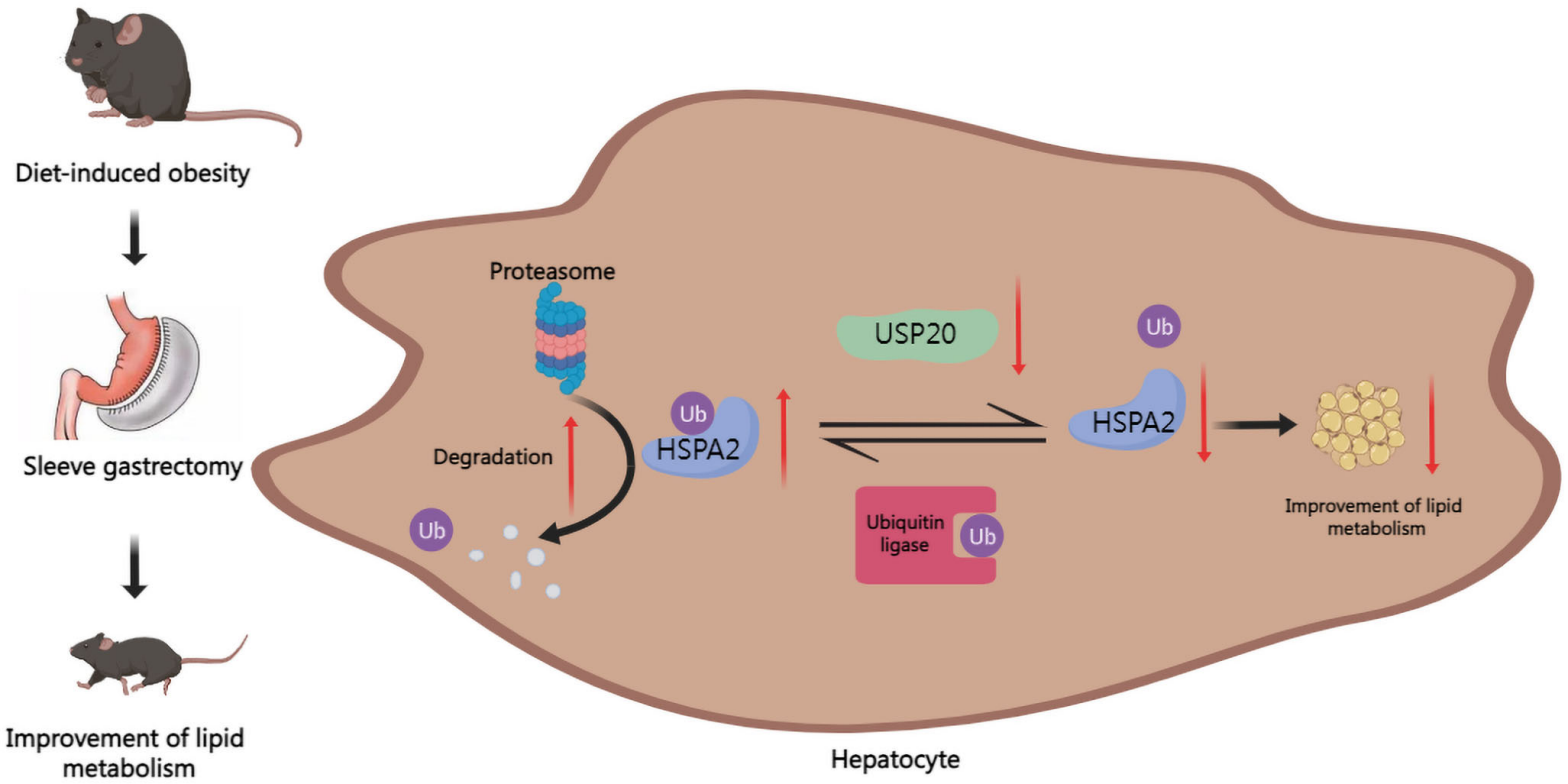
Graph of the potential mechanism of the regulatory network. SG improves lipid dysmetabolism *via* downregulating USP20-HSPA2 axis in DIO mice.

Our results demonstrated that in DIO mice, USP20 is a DUB that targets HSPA2 and stabilizes HSPA2 *via* deubiquitination. It also showed that SG improves lipid metabolism by downregulating the USP20-HSPA2 axis to some degree (**Figure 6**).

## Discussion

Obesity is a serious health concern that causes a great deal of personal and societal issues (1, 2). Although SG results in a considerable decrease in body mass index and an improvement in lipid dysmetabolism, limitations such as operative trauma and surgical risk lead to unpredictable surgical treatment results in obese patients (3, 4). SG improves lipid metabolism in a weight-loss-independent manner, which has been demonstrated by some studies (27, 28). Therefore, it is vital to elucidate the mechanism by which SG improves lipid metabolism to develop a noninvasive therapy. We propose a possible mechanism demonstrated in DIO mice, involving the downregulation of the USP20-HSPA2 axis. It has also been found that USP20 is a DUB that removes ubiquitin linked to HSPA2 and protects mice from lipid dysmetabolism.

We conducted SG on an individual in a DIO mouse model, which is a model that has been recognized worldwide because it simulates the metabolic characteristics and natural history of obesity (29). After the SG, we observed obvious weight reduction and metabolism improvement, thereby demonstrating the success of model establishment. Within the mechanism of lipid metabolism after SG, bile acids and bile acid receptors have attracted great attention (8, 30, 31). Some studies have shown that the improvement in metabolism of SG is associated with the elevation of serum bile acid (27, 32). Several nuclear receptors are activated by bile acids to regulate physiological functions. Farnesoid X receptor (FXR) and Takeda G protein-coupled receptor 5 (TGR5) are two of the most important receptors. According to some studies, cholic acid, a natural FXR ligand, protects the liver from steatosis and treats hyperlipidemia in mice (33). Steroidal FXR agonists have been found to play a beneficial role in nonalcoholic fatty liver disease (34–36). Ding et al. suggested an essential role of TGR5 in reducing liver lipid accumulation after SG (37). However, the downstream mechanisms of bile acid receptors are not completely clear. In eukaryotic cells, the ubiquitin–proteasome system (UPS) is the most noteworthy post-transcriptional modification, which is widely involved in multiple physiological and pathological regulation (38). Some studies have found that bile acid signaling pathways perform several diverse biological functions *via* UPS (39–41). DUBs are proteases that function in the reverse direction of ubiquitination, and mediate deubiquitination (12). More than 90 members of the DUB family are further divided into seven subfamilies, and USP20 belongs to the USP subfamily (14). According to the latest research, USP20 knockout mice present a series of characteristics associated with the improvement of lipid dysmetabolism, which suggests that bile acid likely achieves its function *via* USP20 in SG (20). We observed USP20 downregulation in the SG group and an *in vitro* study confirmed that the expression changes of USP20 regulated lipid metabolism. Further investigation into the relevant pathways and molecular mechanisms is needed.

According to molecular weight, the heat shock protein family is generally divided into six groups: 27-kDa, 40-kDa, 60-kDa, 70-kDa, 90-kDa, and large heat shock proteins (42). The primary functions of heat shock proteins include protein folding, protein complex assembly, protein transportation, and protein degradation (42–44). The 70-kDa heat shock proteins are ubiquitously expressed and are considered to regulate lipid metabolism because of their chaperone properties (44–46). HSPA2 is a 70-kDa heat shock protein (HSP70) that has been identified as a potential substrate of USP20 by MS analysis. Unlike USP20, HSPA2 presents downregulation in the SG group only at the protein level. We also observed HSPA2 promoted lipid accumulation *in vitro*. Further studies found that USP20 positively regulates HSPA2 only at the protein level, which confirms that USP20 plays a vital role in lipid metabolism improvement by targeting HSPA2 after SG. Ding et al. found that the ubiquitin ligase RNF144A suppresses the growth and metastasis of breast cancer by regulating the stability of HSPA2 (47). Furthermore, the circBoule RNA of mouse sperm interacts with HSPA2, and circBoule RNA targets and regulates the stability of HSPA2 *via* ubiquitination (48). These results suggest that the ubiquitin-proteasome pathway is involved in HSPA2 related biological functions. These results were consistent with our findings that USP20 interacted with HSPA2 and stabilized HSPA2 *via* deubiquitination.

Our study identified a new pathway involved in SG where the downregulation of USP20-HSPA2 axis improved lipid metabolism. Zhang et al. showed that the overexpression of HSP70 enhances TG accumulation with the upregulation of fatty acid biosynthesis enzymes, including fatty acid synthase, stearoyl-CoA desaturase and acetyl-CoA carboxylase (49). HSP70 promotes TC accumulation by reducing the expression levels of the ATP-binding cassette transporter A1 and ATP binding cassette transporter G1 (50). Therefore, fatty acid biosynthesis and cholesterol transport are the possible pathways by which the USP20-HSPA2 axis achieves biological functions. Some limitations of this study include the necessity of *in vivo* experiments to verify the effect of the USP20-HSPA2 axis on lipid metabolism. In addition, the intermediate links connecting SG and USP20 still need to be identified. The downstream pathway of the USP20-HSPA2 axis needs further studies for validation. We hope to address these limitations in our future studies.

In conclusion, our study provides evidence that the USP20-HSPA2 axis is downregulated in the SG group. USP20 stabilizes HSPA2 *via* deubiquitination to promote lipid accumulation, which may be a noninvasive therapeutic target for replacing surgery.

## Data availability statement

The data presented in the study are deposited in the PRIDE repository, accession number PXD037693.

## Ethics statement

The animal study was reviewed and approved by the Medical Ethics Committee of Shandong Provincial Qianfoshan Hospital, Shandong University.

## Author contributions

Conceptualization, WZ and MZ. Methodology, WZ, YC, JZ, GZ. Validation, WZ, BS, SRL, ZL, SHL, SD. Data analysis, WZ. Writing original draft preparation, WZ. All authors contributed to the article and approved the submitted version.
