# Supplementary material for: Sleeve gastrectomy improves lipid dysmetabolism by downregulating the USP20-HSPA2 axis in diet-induced obese mice
Source: Front Endocrinol (Lausanne). 2022 Dec 27;13:1041027. doi: 10.3389/fendo.2022.1041027 (PMC9831654; doi:10.3389/fendo.2022.1041027)

Supplementary figure S1. Body weight after HFD (n = 12).

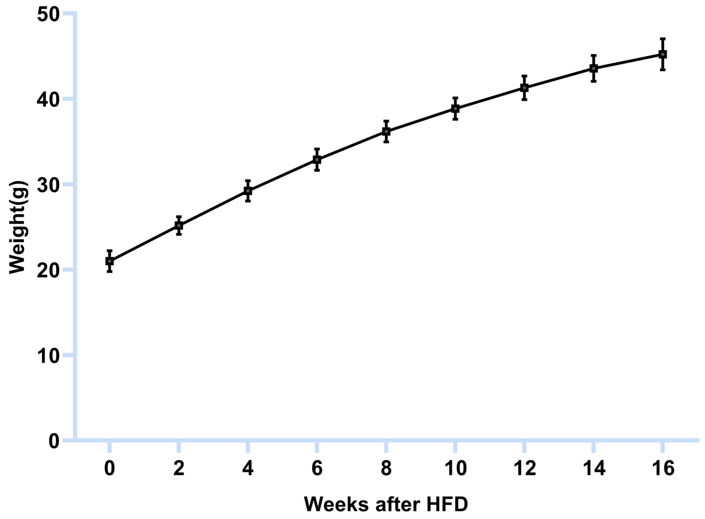

Supplementary figure S2. Serum TG levels of sham and SG group before operation (n = 6). The data are expressed as mean ± SEM, Student's t-test was used for intergroup comparisons.

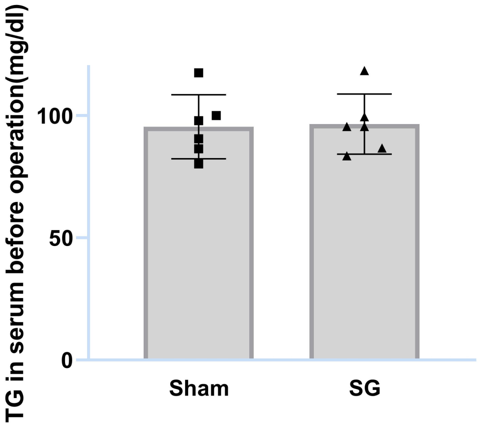

Supplementary figure S3. Serum TC levels of sham and SG group before operation (n = 6). The data are expressed as mean ± SEM, Student's t-test was used for intergroup comparisons.

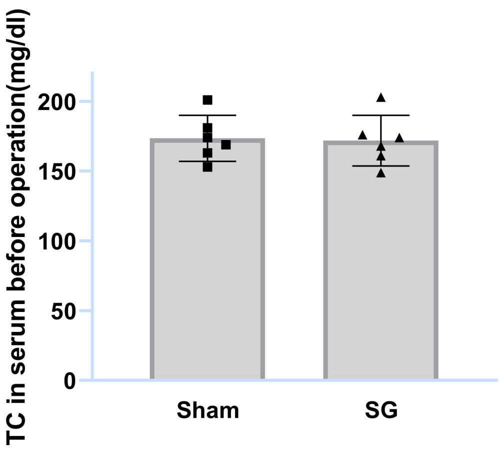

Supplement: Supplementary file 1 [file DataSheet_1.pdf]
